# Book Notices

**Published:** 1894-03

**Authors:** 


### Book Notices.

---

SURGERY: A PRACTICAL TREATISE WITH SPECIAL REFERENCE TO TREATMENT. By C. W. Mansell Moullin, M. A., M. D. Oxon. Fellow of the Royal College of Surgeons; Surgeon and Lecturer on Physiology to the London Hospital; Formerly Radcliffe Traveling Fellow and Fellow of Pembroke College, Oxford, England. Assisted by various writers on special subjects, with six hundred illustrations, many of which are printed in colors, about two hundred having been made from special drawings. Second American edition, revised and edited by John B. Hamilton, M. D., LL. D., Professor of the Principles of Surgery and Clinical Surgery, Rush Medical College, Chicago; Professor of Surgery, Chicago Polyclinic; Surgeon, formerly Supervising Surgeon-General U. S. Marine Hospital Service; Surgeon to Presbyterian Hospital; Consulting Surgeon to St. Joseph's Hospital and Central Free Dispensary, Chicago; Secretary-General of the Ninth International Medical Congress, etc. In one handsome royal octavo volume, of nearly 1250 pages. Price, cloth, $7. P. Blakiston, Son & Co., Publishers, 1012 Walnut street, Philadelphia.

This is one of the very latest and best text books on the Theory and Practice of Surgery. The first edition was introduced into this country about three years ago, and the demand for it was so great that it was soon exhausted, and a new one was printed within two years. In the preparation of this edition, many changes have been made in consequence of the many advances that have been made in surgical science, and quite a number of additions have been made. The author has endeavored to make this book a practical one, as he says, that it may be of greater service to students and practitioners, and with this object before him, he has given special attention to treatment. His plan of including under the head of each organ a "brief description of the malformations to which it is liable and the various operations that may be performed upon it," is quite an improvement over the plan of considering these things under separate chapters. It is much more convenient for the student, and will save him much time to find a full and complete description of the organ, the diseases and injuries to which it is liable, and the treatment, operative or otherwise, all embraced in one chapter, instead of having to search different parts of the volume for these matters, as is necessary in many of our works on surgery.

The book is divided into three parts, Part I being devoted to the general pathology of surgical diseases; Part II, to injuries, and Part III to the diseases and injuries of special structures and organs.

In the preparation of this edition, a thorough revision has been made wherever necessary, and many new illustrations from the most recent foreign and domestic monographs, have been added. A brief chapter on military surgery has been added by the editor.

The excellent general arrangement of the book, its concise and yet thorough presentation, and the large number and high class of its illustrations will largely increase the popularity of this already popular work. H.

SUPPLEMENT TO THE REFERENCE HANDBOOK OF THE MEDICAL SCIENCES, by various writers. Illustrated by Chromo-lithographs and Wood Engravings. Edited by Albert H. Buck, M. D., New York City. William Wood & Co., Publishers, New York.

Volume IX. This volume consists of 1076 pages, closely printed and somewhat larger than the pages of the other eight volumes of the Reference Handbook. It is published for the purpose of bringing the Reference Handbook of the Medical Sciences fully up to date, and this work on the part of the editor and publishers, will be highly appreciated by those physicians especially who possess the other eight volumes of this magnificient work. That it was necessary to revise the Handbook, any physician that has noted the many advances that have been made in medical science, especially in the departments of Pathology, Materia Medica and Therapeutics, will readily agree. The decision to issue a new volume in the form of a supplement rather than to revise the volumes of the work and issue a new edition of the whole' was a wise one, and this decision will receive the approbation of the thousands of physicians who have the Handbook in their libraries. This method of revision will enable present possessors of the work to obtain the new matter at a comparatively small cost.

The same general care has been exercised in the selection of writers for this volume as was observed in the selection of those for the original work, and the list embraces the names of one hundred and twenty of the leading medical men of this country and Canada. Among the list of contributors may be mentioned the names of Dr. Robert Abbe; Dr. John Aulde, Dr. A. D. Blackader, Dr. Joseph D. Bryant, Dr. Henry T. Byford, Dr. N. S. Davis, Dr. F. X. Dercum, Dr. J. McF. Gaston, Dr. John B. Hamilton, Dr. James Nevins Hyde, Dr. William M. Johnston, Dr. William W. Keen, Dr. Chas. B. Kelsey, Dr. Wesley Mills, Dr. Joseph O'Dwyer, Dr. F. Peyre Porcher, Dr. Mary Putnam Jacobi, Dr. A. D. Rockwell, Dr. Louis McLane Tiffany, Dr. A. VanderVeer, Dr. Ely Van DeWarker, Dr. Jas. T. Whittaker, Dr. Burt G. Wilder, Dr. Alfred C. Wood, and many others equally prominent. It is safe to say that few if any works on medical science are so fortunate as to have had the services of so many men of prominence in the profession in its production.

That the editor of this volume and the contributors to it have done their work well, goes without saying. Any physician con templating the purchase of it, may rest assured that it will put him in possession of all the latest facts embraced in the medical sciences, and this without having to wade through a mass of useless matter. H.

---

ANNUAL OF THE UNIVERSAL MEDICAL SCIENCES, A yearly report of the progress of general sanitary sciences throughout the world. Edited by Charles E. Sajous, M. D., and seventy associate editors, assisted by over two hundred corresponding editors, collaborators and correspondents. Illustrated with chromo-lithographs, engravings and maps. Sixth issue, 1893. Price, cloth, $15; half Russia, $20. The F. A. Davis Co., publishers, Philadelphia, New York, Chicago and London.

Sajous' Annual has now become a household word with the physicians of this country. For the past five years the Annual has been a welcome visitor to the offices of the majority of progressive physicians. It has increased in worth and in popularity from year to year, and this, the sixth year, is better than any former edition. A number of changes have been made in the editorial staff, and the book has gained in interest and usefulness as a consequence. The first corps of editors was, as a matter of course, largely an experiment, most of them being authors and men of prominence, but after due trial, in connection with this work, it was found that they were not suited to this work, or did not take sufficient interest in it to make of the Annual that success which the editor desired and expected. From year to year some of the old names have been dropped and new ones added, each of the new men being selected on account of his special fitness for the work he was expected to perform, and his willingness to give the matter the time and labor necessary. With further experience the editor has seen the weak points of the first year's issues and has carefully corrected them, cutting off or adding to wherever the general interest or practical value of the book demanded such changes.

With the sixth year's Annual several prominent European physicians have been placed in charge of departments, viz.: G. Dujordin-Beaumetz, B. W. Richardson, R. Lépine, H. Obersteiner, A. Bourneville, Norman Kerr, G. Apostoli, A. Lutand, D. W. Buxton, P. P. Budin, F. Levison and P. Poirier. These men represent the countries in which, outside of its native land, the Annual has found the greatest appreciation, and their contributions will add much to the value of the work. We predict that this year's Annual will have a larger sale than any previous issue; and when we consider the excellence of the work of the Editor and contributors, the many improvements that have been made over former years, the amount of valuable matter fully up to date, and in a convenient and reliable form, it is rather surprising that any progressive man would attempt to do a general practice without it. H.

---

A TEXT BOOK OF THE THEORY AND PRACTICE OF MEDICINE BY AMERICAN TEACHERS. Edited by William Pepper, M. D., L.L.D., Provost and Professor of the Theory and Practice of Medicine and of Clinical Medicine in the University of Pennsylvania. In two Royal octavo volumes of about 1000 pages each. For sale by subscription only. Price per volume, cloth, $5; sheep, $6; half Russia, $7. W. B. Saunders, Publisher, 925 Walnut street, Philadelphia, Pa. Vol. II.

We are pleased to announce that the second volume of this most excellent work is now ready for distribution. Owing to a number of causes this number has been delayed some time, and the publisher has tendered to each subscriber a good and sufficient reason for its non-appearance at an earlier date, and has expressed his appreciation of the very kind indulgence in this matter by the profession generally.

The JOURNAL gave an extended review of the first volume of this work, some months since, and it is hardly necessary to add more than a word at this time. The second volume comes fully up to the high standard of excellence that characterized the first volume. It is truly a most excellent work, and as near indispensable as any work on the Theory and Practice of Medicine can be. More than three hundred pages of volume second and about two hundred pages of volume first are from the pen of Dr. Pepper whose writings are so popular with the American physicians. A number of other great teachers, Dr. Wm. H. Welch, Dr. Henry M. Lyman, Dr. William Osler, Dr. James C. Wilson, Dr. Francis Delafield, Dr. James W. Holland, and Dr. Reginald H. Fitz have contributed articles to the second volume, and each of them has done his work in the most masterly manner. Dr. Pepper was very fortunate indeed in being able to secure the services of men of such high rank and ability to assist him in bringing out this great text-book. The publisher has done his work in his usual splendid style, and he announces with some pride that the press-work of this volume was done on his own new presses erected for this purpose. H.

MESSRS. WM. WOOD & CO., are pleased to announce that they have in course of publication, and shall publish as early as possible at intervals of four months, a very important work on Medical Jurisprudence, Forensic Medicine and Toxicology, prepared by experts of acknowledged authority, under the supervision and editorship of R. A. Witthaus, M. D., of New York, and Tracy C. Becker, Esq., of Buffalo, N. Y.

This important publication will consist of four large octavo volumes (of from seven hundred to eight hundred pages each), which will be published as rapidly as they can be issued from the press.

These volumes will be printed in the best manner, from new type, and illustrated wherever desirable, by line and "half-tone" engravings and chromo-lithographic plates. In paper, press work and binding they will be samples of the best work.

It will be bound in muslin at $5.00 per volume, and in leather at $6.00 per volume.

Sold by subscription only. Single volumes not sold.

---

AN OPERATION BLANK WITH LISTS OF INSTRUMENTS, ETC., Required in Various Operations. Prepared by W. W. Keen, M. D., LL. D., Professor of Principles of Surgery in the Jefferson Medical College of Philadelphia. Second edition. Price per Pad, containing Blanks for 50 Operations, 50 cents, net. W. B. Saunders, Publisher, 925 Walnut street, Philadelphia.

A convenient blank, suitable for all operations, giving complete instructions regarding necessary preparation of patient, etc., with a full list of dressings and medicines to be obtained from drug store.

At the back of pad is a list of instruments used—viz.: general instruments, etc., required for all operations, and special instruments for surgery of the brain and spine, mouth and throat, abdomen, rectum, male and female genito-urinary organs, the bones, etc., etc.

The whole forming a neat pad arranged for hanging on wall of surgeons office or hospital operating-room.

---

ESSENTIALS OF BACTERIOLOGY—Being a Concise and Systematic Introduction to the Study of Micro-Organism. For the Use of Students and Practitioners. By M. V. Ball, M. D. With eighty-one illustrations, some in colors and fine plates. 200 pages. Price, cloth, $1.00. Philadelphia: W. B. Saunders. 1893.

In this little book Bacteriology is considered under wo heads,

General Consideration and Technique, and Special Bacteriology. It also has an elaborate appendix. The popularity of the book is attested by the appearance of this, the second edition, so soon after the first. As many important changes have been made in keeping with the progress of this branch of medical science, the book will doubtless increase in popularity. H.

---

STERILITY IN THE WOMAN, AND ITS TREATMENT. By Dr. DeSinety. Translated by E. P. Hurd, M. D. Physicians' Leisure Library Series. 130 pages. Price, paper, 25 cents; cloth, 50 cents. George S. Davis, Publisher, Detroit, Mich.

Dr. DeSinety's well-known reputation as a gynecologist is a sufficient guarantee of the merits of the book. Both the author and the translator deserve to be commended for the excellence of their work. H.

---

RECENT DEVELOPMENTS IN MASSAGE, Historical, Physiological, Medical and Surgical. By Douglas Graham, M. D., Boston, Mass. Second Edition. 128 pages, 17 illustrations. Price, paper 25 cents, cloth 50 cents. George S. Davis, Publisher, Detroit, Mich.

With a peculiar introductory chapter, and many other peculiarities throughout the body of the work, this little book abounds in many new and useful suggestions respecting the wide range of usefulness of massage. It is a very sprightly book on a very interesting subject. H.

